# Some Thoughts on Municipal Milk-Inspection1Presented at the annual meeting of the Pennsylvania State Veterinary Medical Association, March 4 and 5, 1902.

**Published:** 1902-06

**Authors:** J. M. Carter

**Affiliations:** Philadelphia


### THE JOURNAL

OF

### COMPARATIVE MEDICINE AND VETERINARY ARCHIVES.

VOL. XXIII. *JUNE, 1902.* No. 6.

### SOME THOUGHTS ON MUNICIPAL MILK-INSPECTION.[1]

BY J. M. CARTER, V.M.D.,
PHILADELPHIA.

FROM my actual and practical experience in the milk business in almost all its various phases, and from my observations as a veterinarian practising in one of the first dairy districts of the country, I have had an opportunity to observe and know pretty nearly the actual character of the milk consumed by the people of a large city like this; and I think I can safely say that if the consumer were to see the cow that produced the milk used on his table and follow that milk from the cow to the table he would use it very sparingly on his oatmeal and in his coffee, and, least of all, give it to his baby or sick child to drink. Of course, there are some well-equipped dairies and careful dairymen who are producing some wholesome milk, but I am speaking of the great majority of dairies as I have seen them. We all know how readily almost all germs grow and develop in milk, and how easily it becomes infected, and being consumed in the uncooked or raw state it must be the medium by which the germs of many infectious and contagious diseases are taken into the system. The history of many epidemics of typhoid fever, scarlet fever, and diphtheria, to say nothing of tuberculosis, can be traced to this source. It is hardly necessary for me to enumerate to you the places and modes in which milk becomes contaminated. It seems to me everything is dirty in connection with milk: the stable, so often dark and damp and poorly ventilated, with little or no

[1] Presented at the annual meeting of the Pennsylvania State Veterinary Medical Association, March 4 and 5, 1902.

drainage—veritable breeding-places for disease; the cows, packed as close as possible to economize room, and stables shut up tight in winter time, to keep each other warm, and in the morning the air is so heavy with ammonia and offensive gases you can hardly breathe. The drinking-water for the cows often comes from barn wells into which drain the barnyard or out-buildings.

At milking times the milk cans are often brought into the stables or entry, and remain there until the milking is finished, in the atmosphere laden with dust and exhalations from the cows; and, in fact, I have often seen the milk cans remain in the stable all night in the winter time, to keep the milk from freezing.

The cows are seldom or never cleaned, and are covered with loose hair and exfoliating epithelium, and their quarters and bellies are plastered with dried or wet effete matter, which is constantly falling into the bucket or being brushed off by the milker, generally with dirty hands and dirty clothes, often wetting the teat with milk or froth, which drips into the bucket; or I have seen the filthy habit of spitting on the hands to wet the teats; in fact, very seldom is any regard whatever paid to cleanliness or hygiene. I have often seen thick milk, stringy milk, bloody milk, all dumped into a can together; and not even when the cow puts her foot in the bucket is the milk seldom rejected.

The buckets and strainers are but little improvement on the other procedures, especially the strainers, which are generally in bad repair, only catching the larger portions of foreign matter which have dropped in, allowing the bulk of the dirt to pass through and settle to the bottom of the can. On arriving in the city in the not overly clean cans the treatment is somewhat better, but even here milk shipped in bulk is subjected to much exposure on the railroad platform. It is often dumped around from one can to another, and sampled and tasted before taking home. Many milk-houses are well equipped, but there are many very badly, and often next to the kitchen or living room, with the doors constantly open.

The manner of handling milk on wagons is especially bad, the cans being opened at every stop and exposed to the dust of the streets and alleys, which is made up largely of dried horse manure and sweepings from houses. Milk in bulk leaves many temptations to the milkman. If a little short of supply, how easy it is to fill up the can with skimmed milk or possibly water, and in the summer time preservatives, which are found in nearly all milk-houses, and in the winter coloring-matter.

One who has never lived in the country or been associated with the dairy business does not know these things, and the majority of consumers of milk look only at the milk as it appears before them, and think only of the Jersey cow, the green fields, the buxom dairymaid, and the old spring-house, with its cool, crystal water. These are the thoughts that present themselves to the ordinary city resident, whose knowledge of the dairy is only his recollections of boyhood days or a week's stay in the country in summer time. Such people only judge of the quality of milk by its color and the amount of cream it raises.

Possibly a great deal of this milk, even if teeming with germs from filth and exposure, is drunk and consumed, and does no apparent harm ; but the manner and carelessness with which the bulk of the milk is handled certainly leaves many ways by which the milk may become contaminated with disease germs and bring serious trouble, far greater, I believe, than is as yet known. We have already some astounding reports from the few observers who have made a study of the spread of diseases through contaminated milk, but we can never know how far-reaching or how great the loss of health and life has been through our pernicious milk-supply.

Dr. Hart and Dr. Freeman, of New York, have collected statistics of thousands of cases of diphtheria, typhoid fever, and scarlet fever traced directly to the milk-supply, the milk becoming infected from the cow producing it or during its handling from the cow to the consumer. Cholera, dysentery, acute milk poisoning, and cholera infantum of children have been traced to milk by the few who have traced the source of these diseases.

Dr. Freeman has classified diseases conveyed by milk to man into three classes :

First, those in which the disease germs are introduced into the milk from the body of the diseased cow ; second, those in which the germs are introduced into the milk from some other source, either during or after milking ; third, those diseases caused by milk which contains poisonous agents developed by bacterial growth.

First. Tuberculosis : We shall consider the diseases under each class separately. By far the most important disease under the first class is tuberculosis. Although disputed by some, the vast amount of evidence and cases recorded — both circumstantial and positive accidental cases — seem to prove beyond a doubt that innumerable cases of tuberculosis in man have been caused directly by consuming milk from tuberculous cows. It is not necessary for me to cite these cases here; they are too familiar to you all.

Second. Anthrax: A few observations of infection by milk from anthrax cows have been made where the disease is more prevalent. In this country, where the disease is comparatively rare and generally of so severe a form and pronounced symptoms, the milk is seldom used—at least, I have no records of such.

Third. Foot-and-mouth-disease; also comparatively unknown in this country. In England the disease has been caused in man directly by consuming milk from cows suffering from the disease.

Acute enteritis: Although the number of cases is small, there does seem to be direct evidence in two or three cases where the disease was traced directly to a cow suffering from disease and transmitting the same to all those drinking her milk. If more observations were made doubtless more cases could be traced to the same source. Under this class may also be considered disturbances often seen in young children, as colic, cramps, vomiting, and diarrhœa. These disturbances are often due to changes in the milk when a cow is suffering from garget or mammitis, and even if the ropy, stringy, often pus-like milk from the affected quarter of the udder is discarded, the remainder of the milk is found to be acid, and has often been known to cause these disturbances when fed to infants. Similar results have been noted on feeding infants on milk from cows unduly excited from any cause, especially at rutting period, when the cow is allowed to race after other cows and often create much excitement in the herd. It is my belief that much of the digestive disturbances of infants is due to improper milk, and that the cow furnishing the milk for the baby should have just as good care and food as a mother suckling her baby.

Certain poisonous plants when eaten by the cow may cause no apparent disturbance to the cow, but transmit the poisonous principle to the milk, which affects infants in different ways, depending upon the character of the plant eaten. The use of milk too soon after calving—before the milk is entirely free from colostrum—causes colicky pains, vomiting, and diarrhœa in infants. Every farmer knows that if you put a six-weeks-old calf on a fresh cow it will cause scours, and yet he does not hesitate to dump her milk into the can after about two or three milkings, when it takes three or four days at least to free the milk from colostrum.

Diseases of the second class, or when the pathogenic organism enters the milk outside the cow: In all epidemics due to milk there are certain characteristics upon which the source of the epidemic is determined, as seldom or never has the germ of the disease been found in the specimen of milk obtained. The cases appear suddenly, there are many new cases each day, and the subsidence is equally marked when the milk-supply is stopped. The houses invaded are widely distributed, and are not restricted to a particular part of the town. The houses of the rich are more apt to be affected than the houses of the poor, as the rich use more milk and often have a special water-supply. The milk drinkers of the family are the ones mostly affected, and the disease is largely among children. In nearly all of the recorded epidemics a patient suffering with the disease has been found at the source in the milk-supply. The most frequent of these epidemics is typhoid fever, of which Dr. Hart, of New York, gives statistics of fifty epidemics, with 3500 cases, and Dr. Freeman gives statistics of fifty-three epidemics and 3226 cases. In my vicinity there have been three epidemics of typhoid fever—one with 50 cases, another 260 cases, and another nearly 100 cases. In the two former, occurring at Elkton, the evidence seems perfect, and was traced directly to the farmer serving the milk, he having typhoid fever in his family and the fever being confined largely to his patrons. , In the latter case, at my own home, although there was typhoid fever on the farm serving the milk to the town, the cases did not confine themselves to his patrons, but existed among the other milkman's patrons furnishing the town as well. As it is known that the milkmen often bought milk from each other, there was some doubt as to the source of the trouble.

Of scarlet fever, forty-one epidemics, with 2393 cases, have been reported. In nearly all of these cases, as in typhoid, a patient suffering with the disease was found at the source of the milk-supply.

Of diphtheria, Dr. Freeman reported eighteen epidemics, with 1000 cases. Not so large a number of the epidemics of diphtheria could be traced to a case of diphtheria on the farm supplying the milk; but with diphtheria in man the attack may be so light as to have been overlooked, or possibly may have been of feline origin, as cats are known to suffer with a throat trouble closely resembling diphtheria, and wheezy old cats are very common around barns, often lapping milk from the buckets and cans. An epidemic of diphtheria has recently occurred in Wayne, in which nearly every case was traced directly to the dairyman's son, who was suffering with the disease.

Cholera epidemics have been traced, through the milk-supply to the farm furnishing the milk, either from a case of cholera occurring on the premises or in the water used to dilute the milk.

In those cases of typhoid fever and scarlet fever where no case existed on the farm it may have been the water-supply on the farm which was polluted, as the barn wells are often drained from barnyards or out-buildings, and it is a common custom for farmers to rinse the buckets and cans in this water and fill the cans with the rinsings.

Disease caused by milk which contains poisonous agents developed by bacterial growth: A few cases of this kind have been recorded in which a large number of people were affected, and on examination the tyrotoxicon of Dr. Vaughn was found in the milk. This poisonous ptomaine is the result of the growth of a certain bacteria which is commonly found in cheese.

In summing up the evidence of the records just given, we may conclude that infection from milk is well established in typhoid fever, scarlet fever, diphtheria, tuberculosis, cholera, foot-and-mouth disease, acute enteritis, and possibly anthrax. Acute milk poisoning in infants, resembling cholera infantum, is frequent, but is seldom traced to the proper source.

Acute cases of poisoning of adults by milk containing tyrotoxicon have occurred. All this is known of the recorded epidemics that have occurred, while many cases are never recorded, and many more are never traced to their source. I believe that all this sickness and death are directly due to contaminated milk, and are to a great extent preventable by proper legislation concerning the inspection of dairies and the handling of the milk which is furnished to our cities and towns.

A study of these epidemics and the evils which have arisen and are arising every day teaches us :

1. That wherever a communicable infectious disease is reported a rigid and careful inquiry into the source of the milk-supply should be made.

2. Proper legislation should be made concerning the inspection of dairies and the handling of milk.

3. In each city or town there should be a special committee or bureau to look after the milk-supply; also to enforce the laws and to have charge of the inspection, and to whom all outbreaks or deaths from contagious diseases should be reported, that the farm and dairy could be examined in search of the cause of the trouble.

4. That each farmer desiring to ship milk for consumption should be compelled to take out a license for the same, and that his dairy, stabling, water-supply, and dairy apparatus should be in a proper condition to produce wholesome milk.

5. There should be sufficient inspectors appointed and paid by the town to which the milk is furnished to inspect each dairy at least every three months, to see that the cows are healthy and that the rules of the bureau are being enforced.

6. That the methods of handling milk in bulk be abolished; that all milk used for food purposes to be consumed raw to be bottled and sealed on the farm, and that seal be not broken until it reaches the consumer, thus avoiding much exposure and any tampering or contamination after leaving the farm; also that each bottle be stamped with the day and date and the name of the shipper.

7. That all whole milk shipped in bulk—or skimmed milk, which is largely used for cooking—if used raw should be heated to at least 155°. The skimmed milk, which largely comes from creameries, is certainly unsafe to feed to a child, as it is submitted to any amount of exposure, and preservatives are used very freely in it. We have all seen too many cases of tuberculosis among calves fed on creamery skimmed milk not to condemn it for infant feeding.

How a people in this enlightened age can go on, year after year, buying and consuming a product produced and marketed with such negligence as regards purity, cleanliness, and healthfulness seems like a relic of barbarism. I believe it is our duty to mankind as veterinarians, and knowing this evil, to expose these facts on all occasions and in every way, and to use every effort to establish the milk-supply of our cities in general on a proper basis; and if we succeed in this it will be one of the biggest and greatest works done to our credit.

